# “Complication unveiled: Bilateral pulmonology embolism in the wake of shoulder arthroplasty” a case report

**DOI:** 10.1016/j.ijscr.2025.111148

**Published:** 2025-03-12

**Authors:** Khalil Awad Almalki, Khalid Jubran Idris, Azzam Ahmed Alzahrani, Salman Ahmed

**Affiliations:** a Department of Orthopedic Surgery, King Abdullah Medical City, Makkah, Saudi Arabia; b Department of Orthopedic Surgery, King Abdullaziz Hospital, Makkah, Saudi Arabia

**Keywords:** Anticoagulant, Computed tomography (CT), Pulmonary embolism (PE), Shoulder arthroplasty (SA)

## Abstract

**Introduction and importance:**

Most common cases of Venous thromboembolism (VTE) seen for those who underwent for lower limb reconstruction and orthopedic trauma surgery in general. VTE was a rare surgical complication after shoulder replacement surgery. This study highlights that pulmonary embolism following upper extremity surgery increased in prevalence without currently guidelines for thromboembolism prevention during upper extremity surgeries especially shoulder arthroplasty surgeries.

**Case presentation:**

80-year-old female patient presented at clinic with right shoulder pain. Clinical features and radiological investigations were suggestive of rotator cuff arthropathy. Following the diagnosis, the patient underwent right shoulder reverse arthroplasty. Two days post operation, the patient exhibited confusion and a decline in consciousness level also decrease in oxygen saturation levels. Clinical features and investigations pointed towards acute pulmonary embolism as the cause of the symptoms. Prompt intervention was initiated, and the patient was started on anticoagulant therapy to manage the condition. With appropriate management, the patient stabilized and was discharged from the hospital.

**Clinical discussion:**

Diagnosis of PE need detailed history and physical examination including vital signs and modality of imaging like spiral Computed Tomography scan, risk factors for pulmonary embolism in patients who underwent shoulder arthroplasty were old age, prolonged surgery, female gender, obesity, diabetes, fluid and electrolyte imbalances, prolonged surgery and undergoing shoulder arthroplasty.

**Conclusion:**

prophylactic anticoagulant therapy should be considered in patients with Risk factor for pulmonary embolism. There is a need to implement strategies, relevant guidelines, and policies to manage high-risk cases with a suspicion of thromboembolism post-shoulder arthroplasty.

## Highlights

• Rare Complication: Bilateral pulmonary embolism is a rare but potentially fatal complication following shoulder arthroplasty.
• Risk Factors Identified: Pre-existing conditions such as obesity, prolonged immobility, and history of thromboembolism increase the risk.
• Diagnostic Tools: CT pulmonary angiography is the gold standard for diagnosing bilateral pulmonary embolism.
• Prevention Strategies: Prophylactic anticoagulation and early mobilization are critical in reducing the risk.

## 1 Introduction

Venous thromboembolism (VTE) presents a well-recognized risk in lower extremity surgeries, such as hip and knee replacement, and orthopedic trauma procedures. Studies indicate that without prophylaxis, the incidence of VTE subsequent to hip and knee arthroplasty which may lead to pulmonary embolism, ranges from 29 % to 60 % [1,2]. In contrast, pulmonary embolism following upper extremity surgery remains infrequent and less documented in medical literature [3]. The first report of VTE after Shoulder arthroplasty (SA) appeared in the literature in 1997 [3] followed by several case reports and retrospective cohort studies in the years to follow. Along with the increasing number of SA procedures performed, reports of VTE after SA have also increased with recent studies, suggesting that those events are not as rare as initially thought. The exact incidence, however, remains unknown [9].

The use of prophylactic anticoagulation in shoulder arthroplasty cases remains a point of contention among surgeons, primarily due to its association with heightened risks of bleeding, wound complications, and the necessity for reoperation. Consequently, the decision to administer prophylaxis hinges significantly on the clinical judgment and suspicion of individual surgeons [3].

After increased the risk of VTE after SA worldwide without established guideline so The purpose of this case report to establish new guideline for prophylaxis of VTE after SA.

## 2 Case report

In this case report, prepared in adherence to the SCARE 2023 guidelines for surgical case reporting [10], a case of 80-year-old female patient, with a medical history significant for diabetes and hypertension, presented complaining of right shoulder pain.

Upon physical examination, no visible deformities or swelling were noted. However, tenderness was observed in the right shoulder area, accompanied by limited range of motion. Several clinical tests, including the Neer impingement test, Hawkin's test, Jobe's test, and drop sign were all positive which indicating possibility of rotator cuff pathology.

**Fig. 1:**
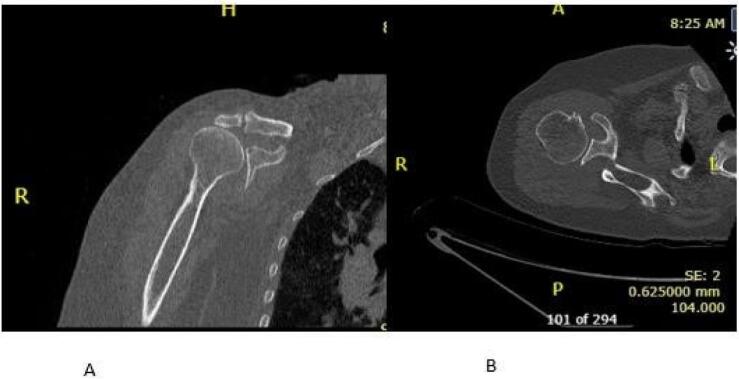
A and B (CT) scan right shoulder: superior subluxation of humeral head, moderate osteoarthritic changes involving glenohumeral joint.

**Fig. 2:**
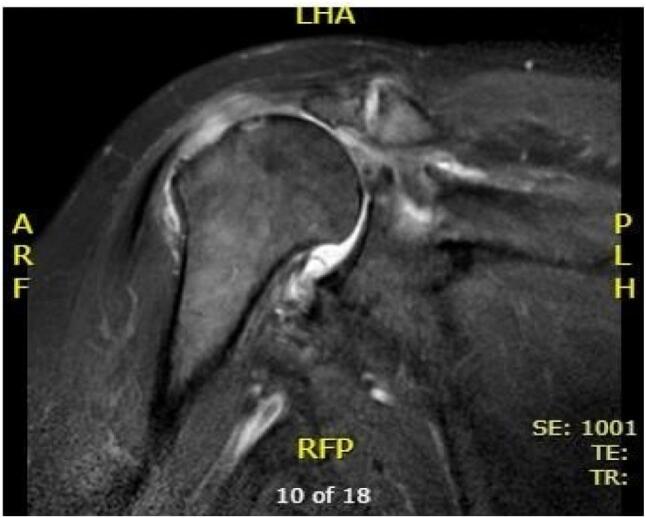
Magnetic resonant image (MRI) showing rotator cuff tear with medial retraction.

**Fig. 3:**
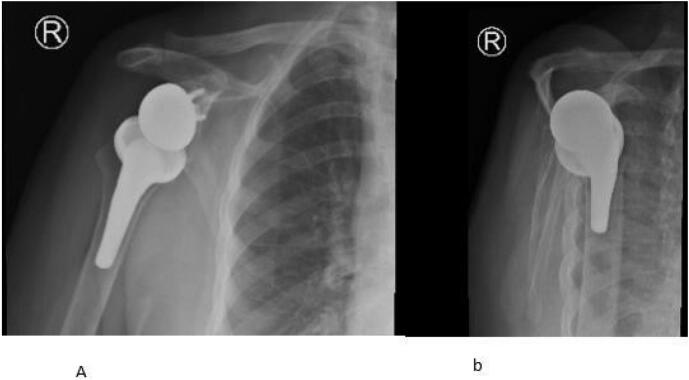
A and B Right reverse shoulder arthroplasty.

Radiological investigations include Computed Tomography (CT) of right shoulder showed superior subluxation of humeral head, moderate osteoarthritic changes involving glenohumeral joint (Fig. 1A and B) and magnetic resonance image (MRI) of right shoulder were suggestive of cuff tear arthropathy (supraspinatus tear with medial tendon retraction, supraspinatus atrophy and arthritis involving glenohumeral joint) (Fig. 2), for which we performed right reverse shoulder arthroplasty (Fig. 3A and B).

First day post operation patient was conscious oriented and vitally stable without chest pain or shortness of breath or signs of deep venus thrombosis. Two days postoperatively, the patient began experiencing confusion and a deterioration in consciousness level, accompanied by a decrease in oxygen saturation. Notably, there were no reports of chest pain or shortness of breath. Vital signs indicated a blood pressure of 139/56 mmHg, a heart rate of 116 beats per minute, a respiratory rate of 20 breaths per minute, and oxygen saturation levels of 90 % on a face mask and 87 % on room air.

Upper and lower limbs examination was unremarkable with no signs of deep venous thrombosis. All the limbs muscles were soft and lax with palpable distal pulses. Given the concerning symptoms, the Rapid Response Team (RRT) was activated. They recommended urgent diagnostic investigations including a Spiral Computed Tomography (CT) scan to rule out pulmonary embolism. The patient was instructed to receive oxygen at a rate of 9 l per minute via a face mask. Additionally, the RRT requested an Echocardiogram (ECHO), serial cardiac enzyme assessments, and a CT scan of the brain.

**Fig. 4:**
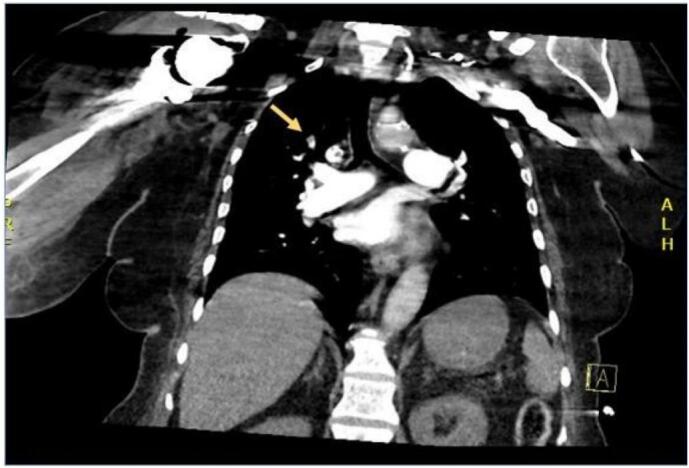
Spiral computed tomography showing right upper segmental lobe filling defect suggestive of pulmonary embolism.

**Fig. 5:**
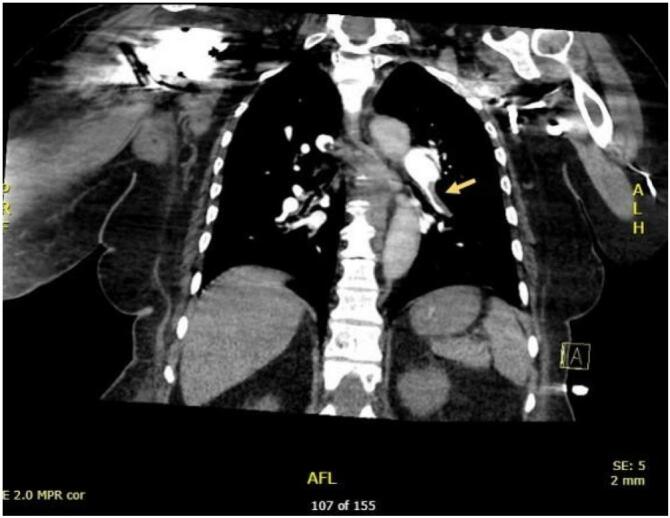
Spiral computed tomography showing left lower lobe filling defect respectively suggestive of pulmonary embolism.

The Spiral CT scan revealed bilateral upper segmental and left lower lobar filling defects consistent with acute pulmonary embolism (Fig. 4, Fig. 5). Consequently, the patient was initiated on a heparin infusion. The ECHO indicated right ventricular strain, while the CT Brain scan showed no evidence of acute insult.

Subsequently, the patient was transferred to the Intensive Care Unit (ICU) for further optimization. The following day the patient underwent catheter-directed therapy. Two days later, the heparin infusion was transitioned to apixaban. Ultimately, the patient was stabilized and was discharged in good condition.

All patient data, including clinical features, investigations, and progress records, were meticulously documented and collected from hospital system for comprehensive management and future reference.

## 3 Discussion

Pulmonary embolism is a major complication of venous thromboembolism [7]. In a study, was found patients with proximal DVT, have associated 40 % with PE, whereas 70 % of patients with PE also have DVT [8].

Most of the researches and studies were reported the incidence of VTE after lower limb surgery. Shoulder arthroplasty (SA) has gained widespread acceptance as a treatment for various shoulder conditions, with annual incidence rates on the rise. [4] Earlier studies have reported that pulmonary embolism (PE) is an uncommon event following shoulder arthroplasty [5], however recent research has highlighted a higher percentage of pulmonary embolism among patients undergoing shoulder arthroplasty [4]. Although the exact incidence is not known, in one study, Sperling et al. found that out of 2885 patients who underwent shoulder arthroplasty, only five developed pulmonary embolism and the time for developing PE was ranged from day of surgery until day seven postoperatively [5]. In another study, out of 31,918 patients who underwent shoulder arthroplasty, 92 patients (0.29 %) developed PE [6].

It has been suggested that the development of DVT is associated with intimal injury, venous stasis, and a hypercoagulable state as a result of related factors within Virchow's triad, this have been postulated to explain the pathogenesis of VTE after SA. It was suggested that during the arthroplasty procedure would cause intimal injury for example direct manipulation, twisting, and stretching of the axillary vein [3].

In a study, the top four predictors for pulmonary embolism in patients who underwent shoulder arthroplasty were proximal humerus fracture, deficiency anemia, congestive heart failure, and chronic lung diseases [7] and none of these were found in our patient.

Other risk factors include advanced age, female gender, obesity, diabetes, fluid and electrolyte imbalances, prolonged surgery (2 to 4 h), and undergoing shoulder arthroplasty rather than hemiarthroplasty [4]. In our case, multiple risk factors such as advanced age (80 years), female gender, obesity (BMI 33), diabetes, prolonged surgical duration (3 h), and shoulder arthroplasty were noted.

VTE prophylaxis was not provided to most patients, likely because of the rarity of the condition and possibly for fear of bleeding complications. When it was used, prophylaxis was mainly mechanical and in the form of TED stockings, pneumatic compression, and early ambulation.

In our case, we did not find any features of deep vein thrombosis on clinical examination of both upper and lower limbs. Radiologically, the absence of deep venous thrombosis was confirmed in the lower limbs by Doppler ultrasound. As our patient was critical and required prompt management, we could not perform Doppler ultrasound for the upper limb to rule out axillary vein thrombosis. Although not very common, it is mentioned in the literature that axillary vein thrombosis can lead to pulmonary embolism [3] and this is one of our limitation in this study.

## 4 Conclusion

Postoperatively, if there is tachycardia and tachypnea with falling oxygen saturation levels, there should be a high degree of suspicion for pulmonary embolism. This should lead to a thorough physical and radiological examination to rule out any pulmonary embolism. Moreover, pulmonary embolism can be fatal, so prophylactic anticoagulant therapy should be considered in patients with advanced age, comorbidities, and those undergoing prolonged surgery. There is a need to advocate and implement strategies, relevant guidelines. Also, we recommend to assess the risk status and using mechanical prophylaxis in all patients undergoing Shoulder arthroplasty.

## CRediT authorship contribution statement

Khalil Almalki (study design)

khalid Idris, MBBS (write a paper)

Azzam Alzahrani, MBBS (write a paper)

Salman Ahmed, md (study design)

## Consent

waiver of consent for participation in this study was requested. Any identifiable patient information was not collected and in this case study did not involve the disclosure of any data that could reveal participant identity and this request was approved.

## Ethical approval

The study protocol was reviewed and approved.

## Guarantor

Dr. Khalil almalki

## Funding

No any source of funding was received.

## Declaration of competing interest

The authors declare that there is no benefit or funds were associated with this study, and there are no conflicts of interest.
